# Beyond omics: From descriptive profiling to causal and scalable design of fermentation microbiomes

**DOI:** 10.1002/imt2.70161

**Published:** 2026-07-30

**Authors:** Dongze Niu, Huili Pang, Xuekai Wang, Liang Song, Jing Zhang, Ansah Terry, Junfeng Li, Jingwen Cao, Qinhua Liu, Dayong Han, Jidong Wang, Jong Geun Kim, Kazuo Ataku, Smerjai Bureenok, Zhongfang Tan, Fengyan Bai, Tao Shao, Taoli Huhe, Lifeng Wang, Peijie Han, Chuncheng Xu, Fuyu Yang, Yimin Cai

**Affiliations:** ^1^ National‐Local Joint Engineering Research Center of Biomass Refining and High‐Quality Utilization, Institute of Urban and Rural Mining Changzhou University Changzhou China; ^2^ State Key Laboratory of Cotton Bio‐breeding and Integrated Utilization, School of Agriculture and Biomanufacturing Zhengzhou University Zhengzhou China; ^3^ College of Grassland Science and Technology China Agricultural University Beijing China; ^4^ Institute of Microbiology Chinese Academy of Sciences Beijing China; ^5^ College of Food Science & Engineering Nanjing University of Finance and Economics Nanjing China; ^6^ Faculty of Agriculture, Food and Consumer Sciences University for Development Studies Tamale Ghana; ^7^ College of Agro‐Grassland Science Nanjing Agricultural University Nanjing China; ^8^ School of Agriculture and Biology Shanghai Jiao Tong University Shanghai China; ^9^ Faculty of Animal Science and Technology Yunnan Agricultural University Kunming China; ^10^ Institute of Green Bio Science & Technology Seoul National University Pyeongchang Korea; ^11^ Department of Dairy Science Rakuno Gakuen University Hokkaido Japan; ^12^ Faculty of Agricultural Innovation and Technology Rajamangala University of Technology Isan Nakhon Ratchasima Thailand; ^13^ College of Engineering China Agricultural University Beijing China; ^14^ School of Animal Science Guizhou University Guiyang China

## Abstract

Fermentation microbiomes play essential roles in food production, feed preservation, waste valorization, and diverse sustainable industrial processes. Although multi‐omics and systems biology have substantially advanced our understanding of their assembly, interactions, and functional dynamics, industrial translation remains constrained by fragmented datasets, limited causal validation, and transport constraints during scale‐up. Large language models act as upper‐level knowledge and workflow orchestrators, accelerating data integration, hybrid AI‐mechanistic modeling, hypothesis generation, and perturbation‐guided learning. Collectively, these advances enable fermentation microbiome research to move beyond descriptive omics toward mechanism‐guided synthetic microbial community design, causal validation, and scalable biomanufacturing.

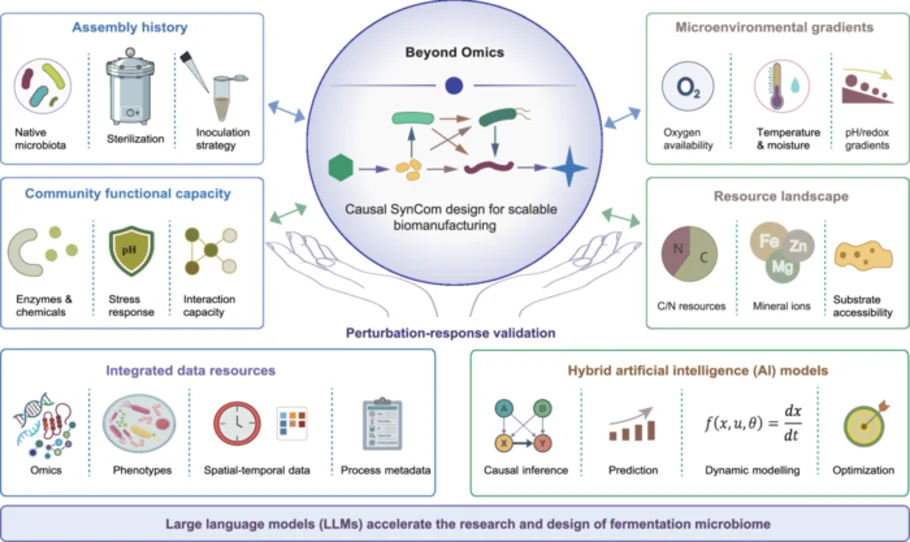

## AUTHOR CONTRIBUTIONS

**Dongze Niu**: Conceptualization; investigation; visualization; writing—original draft; writing—review and editing; funding acquisition. **Huili Pang**: Conceptualization; investigation; visualization; writing—original draft; writing—review and editing; funding acquisition. **Xuekai Wang**: Conceptualization; investigation; visualization; writing—original draft; writing—review and editing; funding acquisition. **Liang Song**: Conceptualization; investigation; visualization; writing—original draft; writing—review and editing. **Jing Zhang**: Conceptualization; investigation; visualization; writing—original draft; writing—review and editing. **Ansah Terry**: Writing—review and editing. **Junfeng Li**: Investigation; visualization. **Jingwen Cao**: Investigation; visualization. **Qinhua Liu**: Investigation; visualization; funding acquisition. **Dayong Han**: Investigation; visualization. **Jidong Wang**: Investigation; visualization. **Jong Geun Kim**: Writing—review and editing. **Kazuo Ataku**: Writing—review and editing. **Smerjai Bureenok**: Writing—review and editing. **Zhongfang Tan**: Writing—review and editing. **Fengyan Bai**: Conceptualization; supervision; project administration; writing—review and editing. **Tao Shao**: Writing—review and editing; supervision; project administration. **Taoli Huhe**: Conceptualization; supervision; project administration; writing—review and editing; funding acquisition. **Lifeng Wang**: Conceptualization; supervision; project administration; writing—review and editing. **Peijie Han**: Conceptualization; supervision; project administration; writing—review and editing. **Chuncheng Xu**: Conceptualization; supervision; project administration; writing—review and editing; funding acquisition. **Fuyu Yang**: Conceptualization; supervision; project administration; writing—review and editing. **Yimin Cai**: Conceptualization; supervision; project administration; writing—review and editing; funding acquisition.

## CONFLICT OF INTEREST STATEMENT

The authors declared no conflicts of interest.

## ETHICS STATEMENT

No animals or humans were involved in this study.

To the Editor,

Fermentation is a process driven by microorganisms and has long been used to preserve food and feed, as well as to produce useful and flavorful compounds [1, 2]. Beyond these traditional applications, fermentation has strong potential for converting plant materials like lignocellulose into valuable products [3, 4]. Although this microbial system usually contains fewer types of microorganisms than natural ecosystems, they often have large microbial populations with high activity, which allows efficient production of important compounds for preservation and industrial use [5, 6].

Compared to well‐mixed industrial liquid fermentation (Figure 1A), high‐solids fermentations, including many semi‐solid and solid‐state processes, under open or semi‐open conditions behave more like spatially structured microbial ecosystems (Figure 1B), where transport limitations and microbial interactions jointly create distinct scale‐up bottlenecks [7]. System behavior is driven not only by complex microbial interactions but also by severe transport limitations, which generate pronounced spatial heterogeneity and multiple environmental hurdles [8]. Over the past decades, research tools have improved from simple observation methods to advanced integrated approaches such as multi‐omics. These advances have greatly improved our understanding of how fermentation systems work and change over time [5, 9, 10], and have supported new processes like converting plant biomass into short‐chain fatty acids [4].

**FIGURE 1:**
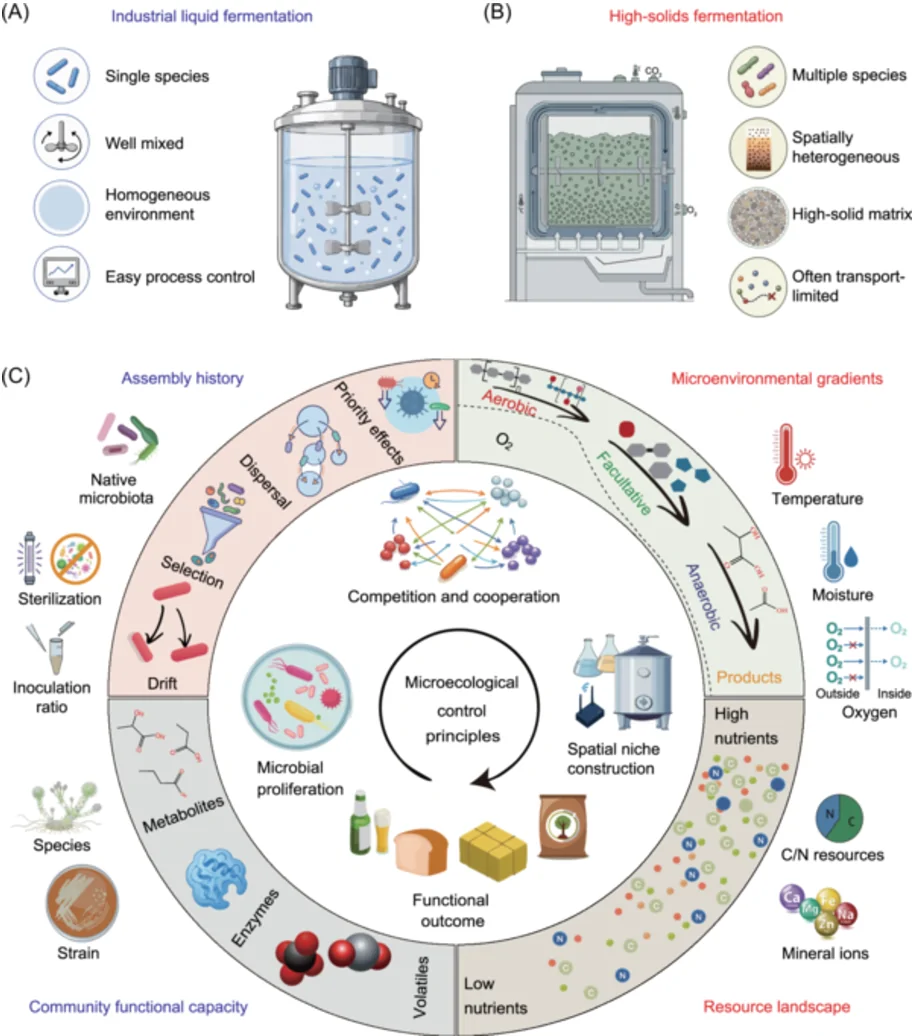
Characteristics of different fermentation systems and microecological control principles in high‐solids fermentation. (A) Industrial liquid fermentation is typically well mixed, relatively homogeneous, and easier to control. (B) High‐solids fermentation under open or semi‐open conditions is characterized by multispecies interactions, spatially heterogeneous substrates, and transport‐limited microenvironments, which reduce predictability and controllability during scale‐up. (C) Microecological control integrates assembly‐guided community design with niche and resource engineering. Assembly history and community functional capacity regulate microbial colonization, selection, dispersal, drift, interactions, and functional outputs, whereas microenvironmental gradients and resource landscapes shape spatial niche construction, metabolic differentiation, and process controllability.

Despite these rapid advances in study methods and microbial ecology, the rational design of fermentation microbiomes for predictable, controllable, and scalable bioprocesses remains a major challenge. This challenge arises because descriptive multi‐omics alone cannot fully explain how scale‐up‐related physicochemical constraints, especially mass and heat transfer limitations, reshape local microenvironments, alter microbial interactions, and limit process controllability. Therefore, we propose a causal, transport‐aware framework in which fermentation microbiomes are treated not merely as communities to be profiled, but as spatially structured, partially domesticated ecosystems to be tested, modeled, and controlled.

## COMPLEXITY AND ENGINEERED DOMESTICATION OF FERMENTATION SYSTEMS

Historically, fermentation evolved from the accidental discovery of spontaneous preservation into a strategically controlled process driven by microbial communities [1, 9]. Through empirical practice and scientific inquiry, humans have learned to control key environmental factors such as temperature, pH, moisture, and substrate levels to guide microbial communities toward desired outcomes [7, 11]. This historical progression reflects a gradient of engineered domestication, transitioning from minimally controlled, open systems (e.g., traditional silage and composting) to more structured, semi‐open processes, such as the solid‐state fermentation of Chinese baijiu.

However, the industrial scalability of these systems varies drastically based on their inherent metabolic and transport demands. Processes like ensiling and composting readily scale because they rely on self‐organizing, low‐oxygen environments with minimal reliance on active heat or mass transfer. Conversely, most aerobic fermentations that demand continuous oxygen supply, heat dissipation, and intensive mixing face severe transport limitations, particularly under high‐solids conditions [8]. These physical bottlenecks compound operational complexity and restrict volumetric scale‐up; therefore, apart from aerobic composting, the single‐unit scale of most high‐solids aerobic fermentations remains highly limited.

To advance beyond these scale‐up bottlenecks, the field must fundamentally reframe its perspective on spatial heterogeneity. Rather than treating transport gradients merely as physical liabilities, we propose that they can serve as powerful design levers. The spatial heterogeneity generated by restricted mass transfer can be intentionally exploited to construct distinct ecological niches and orchestrate targeted microbial interactions, thereby enabling more efficient biomanufacturing strategies [4]. Therefore, fermentation systems are not strictly engineered bioprocesses; they remain partially domesticated ecosystems. In this context, engineered domestication transcends conventional process control or starter culture selection—it is the deliberate shaping of ecological boundaries, spatial niches, and evolutionary selection pressures that dictate microbial community assembly and macroscopic function.

### MICROECOLOGICAL PRINCIPLES AND SPATIAL NICHE CONSTRUCTION

Recent advances in high‐throughput sequencing have substantially elevated microecological theory in fermentation systems by characterizing microbial composition, metabolic traits, and interspecies interactions [7, 10, 12]. While these biotic features provide essential baseline information, they do not fully capture the dynamic, process‐level drivers that govern community assembly (Figure 1C). In practical fermentation design, priority effects, defined as the order and timing of species arrival, and initial inoculation ratios are particularly important because they can determine early colonization, community succession, and metabolic function [13, 14].

As fermentation progresses, abiotic constraints become equally critical. In fact, these physicochemical parameters often function as multiple environmental hurdles, such as simultaneous gradients in oxygen, moisture, pH, and temperature, that act as first‐order determinants of community organization [3, 8, 15]. Particularly in high‐solids and deep‐bed solid‐state fermentations, these multi‐dimensional hurdles drive pronounced spatial heterogeneity [7, 8]. This physical stratification establishes distinct ecological niches and localized interaction networks, which are essential for maintaining the overall resilience and functional differentiation of the microbial ecosystem.

For instance, the spatial separation between kefir grains and the surrounding milk matrix promotes notable spatiotemporal niche differentiation, thereby sustaining community resilience [12]. Similarly, oxygen gradients generated in a membrane bioreactor have been exploited to create distinct spatial niches for different functional groups of microorganisms, thereby enabling the conversion of lignocellulose into short‐chain fatty acids [4]. Therefore, rational process design must advance beyond merely mapping functional potential to actively programming the spatiotemporal heterogeneity of the fermentation environment. This paradigm shift is especially critical during industrial scale‐up, where transport constraints and microenvironmental engineering ultimately determine process stability. In this sense, spatial niche construction, driven by strategically applied environmental hurdles, should be recognized as an experimentally tractable route for improving the predictability and controllability of engineered microbiomes.

### FROM OMICS INFERENCE TO PERTURBATION‐BASED VALIDATION

While genomic profiling outlines microbial composition and functional potential, advanced multi‐omics, spanning transcriptomics, proteomics, and metabolomics, provides critical insights into realized metabolic activities and interspecies exchanges [5, 12, 15]. When coupled with spatially resolved sampling, these tools can effectively map in situ expression patterns across heterogeneous fermentation microenvironments [15]. However, it is crucial to recognize that such omics‐derived networks fundamentally represent correlative, mechanistic hypotheses rather than direct causal evidence.

To bridge the gap between correlation and causation, descriptive omics must be coupled with targeted perturbation. For example, in kefir fermentation, multi‐omics and genome‐scale modeling initially mapped dynamic metabolic cooperation. Crucially, it was subsequent perturbation experiments, such as spent‐medium assays, metabolite supplementation, and pairwise co‐cultures, that causally validated the cross‐feeding of amino acids and vitamins between bacteria and yeasts, confirming that spatial organization underpins community stability [12]. Similarly, in cheese rind microbiomes, multi‐omics combined with high‐throughput fitness screening identified candidate genes driving inter‐kingdom interactions. However, the definitive mechanisms, such as biotin competition and siderophore‐mediated dynamics, were only confirmed through targeted mutagenesis, competition assays, and in vitro validation [16].

These elegant studies demonstrate that multi‐omics is most powerful when embedded within an iterative validation framework: omics identifies candidate interactions, while controlled perturbations directly test their causality. Ultimately, it is this validated mechanistic knowledge, rather than mere compositional correlation, that must serve as the blueprint for the rational, bottom‐up design of synthetic microbial communities (SynComs).

### FROM FRAGMENTED DATA TO REUSABLE FERMENTATION KNOWLEDGE

While omics technologies have fundamentally transformed our understanding of fermentation microbiomes, the field remains constrained by fragmented, system‐specific studies with limited sample sizes [5, 17]. Even in extensively investigated systems like traditional baijiu fermentation, research is frequently confined to specific batches or regional practices [1, 11]. Consequently, cross‐system comparisons are notoriously difficult, hindering the extraction of transferable ecological principles and predictive design rules.

Recent large‐scale integration efforts have begun addressing this bottleneck by standardizing food microbiome data at a global scale (Figure 2A) [5, 17]. For example, the curated Food Microbiome Data recently reconstructed high‐quality microbial and eukaryotic genomes, successfully unveiling cross‐kingdom interactions and linking food‐associated microbiomes to gut health [5]. However, transforming passive data repositories into reusable knowledge systems demands more than mere genomic aggregation. It requires comprehensive multi‐omics coverage, high‐resolution functional annotations, and, crucially, standardized process‐level metadata [5, 17].

**FIGURE 2:**
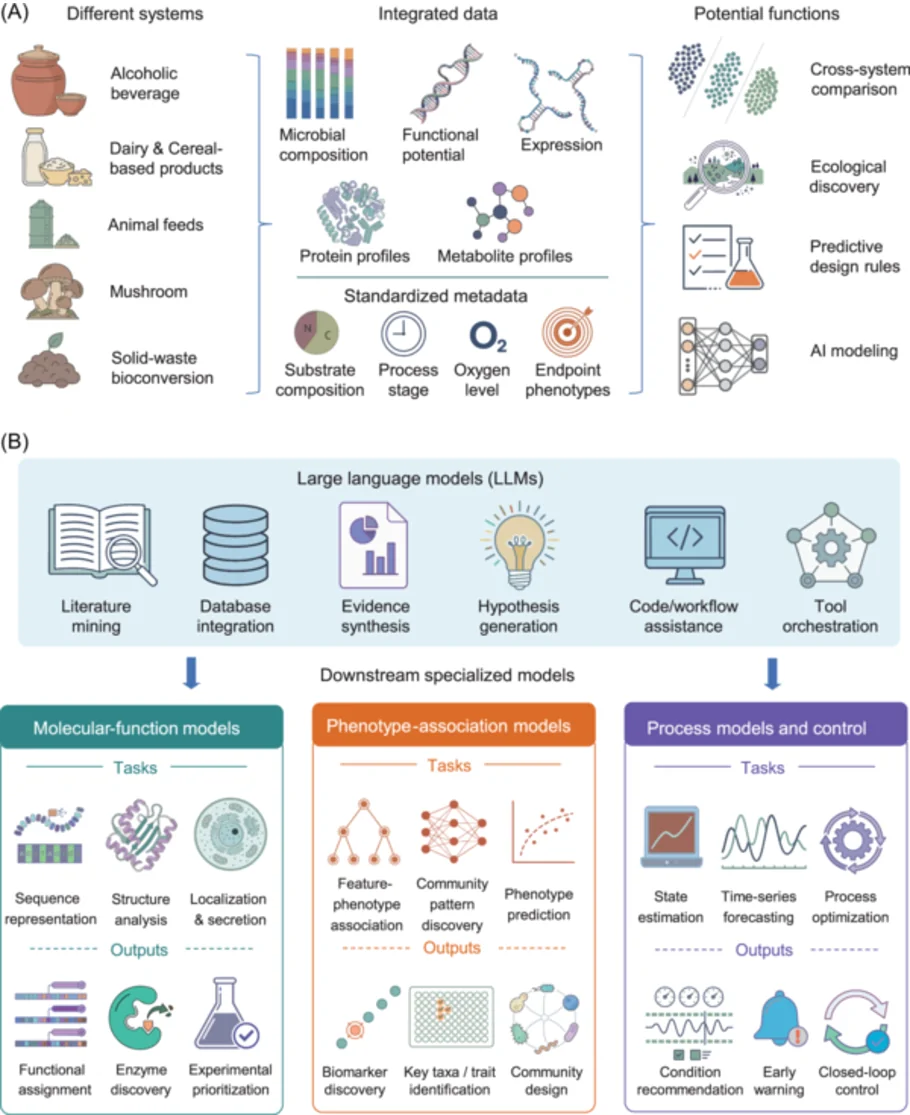
A data‐driven computational framework for scalable fermentation design. (A) Integration of multi‐omics data and standardized metadata across diverse fermentation systems enables the accumulation of reusable knowledge. (B) A hierarchical AI framework for fermentation microbiome research. Large language models (LLMs) function as upper‐level knowledge orchestrators for literature mining, evidence synthesis, hypothesis generation, and workflow coordination, whereas downstream specialized models support molecular‐function analysis, phenotype association, and process control.

For engineered fermentations, biological data must be inextricably linked to physicochemical constraints. Standardized metadata must capture not only sequencing details but also key process variables, such as substrate composition, fermentation stage, and oxygen dynamics [5]. Furthermore, rigorously recording endpoint phenotypes, including product yield, flavor profiles, degradation efficiency, or biomass conversion rates, is paramount. These phenotypic metrics are essential because they define the mathematical objective functions required for predictive modeling and artificial intelligence (AI) assisted optimization. Ultimately, the fidelity and completeness of this metadata dictate the reliability of cross‐system data integration. By anchoring multi‐omics profiles to stringent process parameters and phenotypic outcomes, fragmented datasets can be forged into a unified, machine‐readable knowledge base—the critical prerequisite for driving scalable, AI‐directed fermentation design.

### A MULTI‐LAYERED AI ARCHITECTURE FOR SCALABLE FERMENTATION DESIGN

The rapid emergence of large language models (LLMs) presents transformative opportunities for fermentation microbiome research and related biomanufacturing fields. Their profound value lies not in producing new experimental evidence, but in acting as upper‐level knowledge interfaces that orchestrate literature mining, evidence synthesis, hypothesis generation, and computational workflow automation (Figure 2B). By lowering the technical barriers to complex data analysis, LLMs can coordinate the execution of three essential downstream, domain‐specific components.

First, at the basal layer of molecular‐function modeling, deep biological foundation models, integrating sequence, structure, and multi‐omics data, become indispensable. These models decode complex microbial interactions, predict subcellular localization, and prioritize poorly annotated enzymes, thereby mapping the functional “dark matter” of fermentation microbiomes [18]. Second, to bridge this localized microbial activity with macroscopic outcomes, phenotype‐association models employ advanced machine learning architectures to integrate microbiome profiles and multi‐omics data with standardized process metadata [18, 19]. By linking microbial signatures to endpoint traits, these models drive robust biomarker discovery and accurate phenotype prediction. Finally, the translation of biological potential into manufacturing reality requires engineering and process models that connect microbial functions with operational decision‐making. At this applied stage, time‐series algorithms, soft sensors, digital twins, and optimization frameworks integrate physicochemical parameters, online sensor signals, and historical batch records to support anomaly warning, dynamic fermentation‐state prediction, and feedback‐oriented process regulation.

Nevertheless, computational predictions alone are insufficient for revealing causal mechanisms or guiding scalable fermentation design. To improve their reliability and translational value, in silico discoveries and model‐guided recommendations should be supported by explainable artificial intelligence (XAI) and perturbation‐based validation. XAI can help trace model outputs back to plausible features, drivers, or mechanisms, while biochemical, ecological, and process‐level perturbations provide the causal tests needed to distinguish robust mechanisms from black‐box associations [20]. This coupling between interpretation and validation is particularly important when multi‐layered computational insights are translated into high‐solids and deep‐bed solid‐state fermentations, where severe mass, heat, and momentum transfer limitations strongly constrain scalability [8, 15]. Although modular bioreactor designs can partially mitigate these physical bottlenecks, they inevitably escalate operational complexity and labor demands. A more powerful strategy is to integrate AI‐driven prediction, explainable diagnostics, automated online sensing, and real‐time feedback control, so that the spatial heterogeneity of high‐solids fermentation systems can be actively programmed rather than passively endured. Ultimately, this convergence of machine intelligence and bioprocess engineering realizes the true vision of engineered domestication—transforming empirically managed, labor‐intensive cultures into scalable, energy‐efficient, and deterministically controllable biomanufacturing ecosystems.

Advances in multi‐omics, systems biology, and computational modeling are reshaping our understanding of fermentation microbiomes, moving the field from descriptive profiling toward deeper insights into microbial interactions, spatial organization, and metabolic functions. However, fermentation microbiome research has generated extensive omics resources, yet mechanistic understanding remains comparatively limited. To translate biological discoveries into robust and scalable biomanufacturing solutions, the field must move beyond passive observation and develop causal, transport‐aware design principles that explicitly connect microecological theory with macroscopic bioprocess engineering. A multi‐layered AI architecture can provide an effective computational bridge for this transition. In this framework, LLMs act as upper‐level knowledge and workflow orchestrators, whereas downstream specialized models support molecular‐function analysis, phenotype association, and process prediction and control. When coupled with XAI, perturbation‐based validation, standardized data resources, and rational bioreactor design, this framework can link fragmented omics data, biological mechanisms, and engineering decisions more effectively. Ultimately, this convergence may realize a practical form of engineered domestication, transforming empirically managed microbial cultures into more predictable, scalable, and controllable biomanufacturing ecosystems.

## Data Availability

No new data or code were generated in this review. Supplementary materials (graphical abstract, slides, videos, Chinese translated version and update materials) may be found in the online DOI or iMeta Science http://www.imeta.science/. The data that support the findings of this study are available on request from the corresponding author. The data are not publicly available due to privacy or ethical restrictions.
